# Supplementary material for: First Characterization of a Cyanobacterial Xi-Class Glutathione S-Transferase in Synechocystis PCC 6803
Source: Antioxidants (Basel). 2024 Dec 20;13(12):1577. doi: 10.3390/antiox13121577 (PMC11673678; doi:10.3390/antiox13121577)
Supplement: Supplementary file 1 [file antioxidants-13-01577-s001.zip › Fig S1.pptx]

## Slide 1
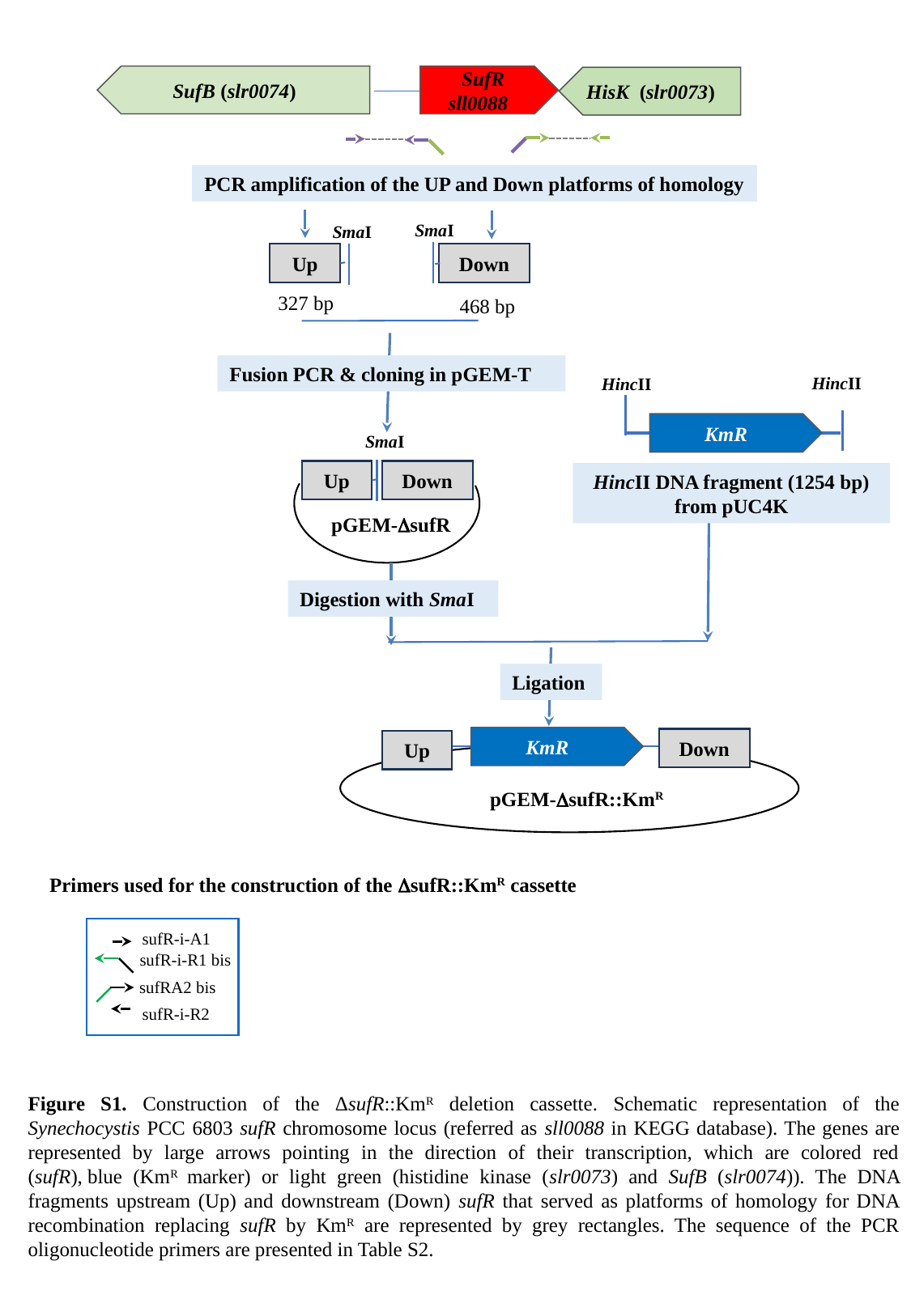

SufB (slr0074)
SufR sll0088
HisK (slr0073)
PCR amplification of the UP and Down platforms of homology
SmaI
SmaI
Up
Down
327 bp
468 bp
Fusion PCR & cloning in pGEM-T
HincII
HincII
KmR
SmaI
Up
Down
HincII DNA fragment (1254 bp) from pUC4K
pGEM-DsufR
Digestion with SmaI
Ligation
KmR
Down
Up
pGEM-DsufR::KmR
Primers used for the construction of the DsufR::KmR cassette
sufR-i-A1
sufR-i-R1 bis
sufRA2 bis
sufR-i-R2
Figure S1. Construction of the ΔsufR::KmR deletion cassette. Schematic representation of the Synechocystis PCC 6803 sufR chromosome locus (referred as sll0088 in KEGG database). The genes are represented by large arrows pointing in the direction of their transcription, which are colored red (sufR), blue (KmR marker) or light green (histidine kinase (slr0073) and SufB (slr0074)). The DNA fragments upstream (Up) and downstream (Down) sufR that served as platforms of homology for DNA recombination replacing sufR by KmR are represented by grey rectangles. The sequence of the PCR oligonucleotide primers are presented in Table S2.
