# Supplementary material for: First Characterization of a Cyanobacterial Xi-Class Glutathione S-Transferase in Synechocystis PCC 6803
Source: Antioxidants (Basel). 2024 Dec 20;13(12):1577. doi: 10.3390/antiox13121577 (PMC11673678; doi:10.3390/antiox13121577)
Supplement: Supplementary file 1 [file antioxidants-13-01577-s001.zip › Fig S2.pptx]

## Slide 1
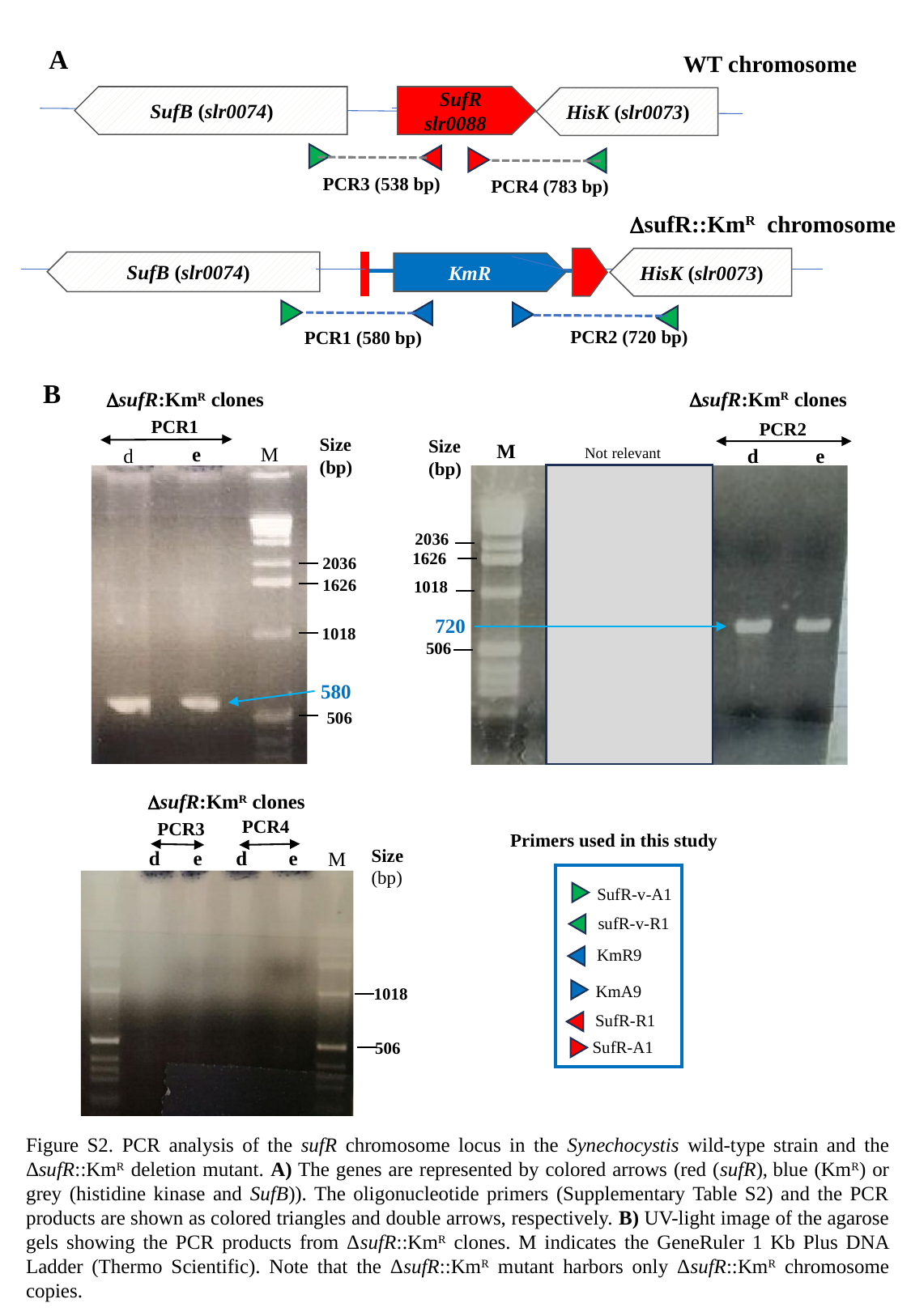

A
WT chromosome
SufB (slr0074)
SufR slr0088
HisK (slr0073)
PCR3 (538 bp)
PCR4 (783 bp)
DsufR::KmR chromosome
HisK (slr0073)
SufB (slr0074)
KmR
PCR2 (720 bp)
PCR1 (580 bp)
B
DsufR:KmR clones
DsufR:KmR clones
PCR1
PCR2
Size
(bp)
Size
(bp)
M
e
M
Not relevant
d
d
e
2036
1626
2036
1626
1018
580
 506
1018
720
 506
DsufR:KmR clones
PCR4
PCR3
Primers used in this study
Size
(bp)
d
e
d
e
M
SufR-v-A1
KmR9
KmA9
SufR-R1
SufR-A1
sufR-v-R1
1018
 506
Figure S2. PCR analysis of the sufR chromosome locus in the Synechocystis wild-type strain and the ΔsufR::KmR deletion mutant. A) The genes are represented by colored arrows (red (sufR), blue (KmR) or grey (histidine kinase and SufB)). The oligonucleotide primers (Supplementary Table S2) and the PCR products are shown as colored triangles and double arrows, respectively. B) UV-light image of the agarose gels showing the PCR products from ΔsufR::KmR clones. M indicates the GeneRuler 1 Kb Plus DNA Ladder (Thermo Scientific). Note that the ΔsufR::KmR mutant harbors only ΔsufR::KmR chromosome copies.
