# Supplementary material for: First Characterization of a Cyanobacterial Xi-Class Glutathione S-Transferase in Synechocystis PCC 6803
Source: Antioxidants (Basel). 2024 Dec 20;13(12):1577. doi: 10.3390/antiox13121577 (PMC11673678; doi:10.3390/antiox13121577)
Supplement: Supplementary file 1 [file antioxidants-13-01577-s001.zip › Fig S3.pptx]

## Slide 1
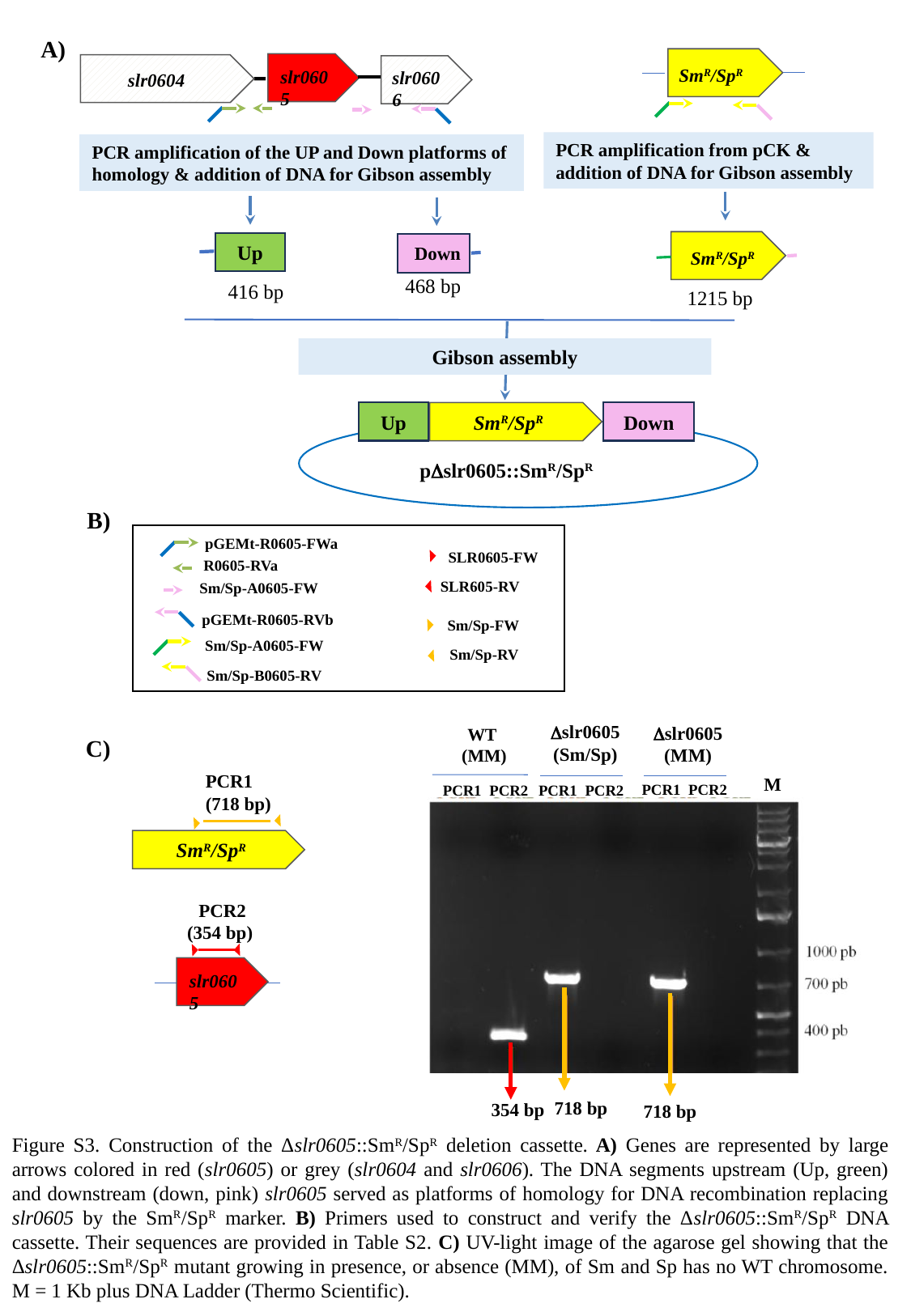

A)
slr0604
SmR/SpR
slr0605
slr0606
PCR amplification from pCK &
addition of DNA for Gibson assembly
PCR amplification of the UP and Down platforms of homology & addition of DNA for Gibson assembly
Up
Down
SmR/SpR
468 bp
416 bp
1215 bp
Gibson assembly
Up
SmR/SpR
Down
pDslr0605::SmR/SpR
B)
SLR0605-FW
pGEMt-R0605-FWa
R0605-RVa
SLR605-RV
Sm/Sp-FW
Sm/Sp-RV
Sm/Sp-A0605-FW
pGEMt-R0605-RVb
Sm/Sp-A0605-FW
Sm/Sp-B0605-RV
Dslr0605
(Sm/Sp)
Dslr0605
(MM)
WT
(MM)
C)
PCR1
(718 bp)
M
PCR1 PCR2
PCR1 PCR2
PCR1 PCR2
SmR/SpR
PCR2
(354 bp)
slr0605
718 bp
354 bp
718 bp
Figure S3. Construction of the Δslr0605::SmR/SpR deletion cassette. A) Genes are represented by large arrows colored in red (slr0605) or grey (slr0604 and slr0606). The DNA segments upstream (Up, green) and downstream (down, pink) slr0605 served as platforms of homology for DNA recombination replacing slr0605 by the SmR/SpR marker. B) Primers used to construct and verify the Δslr0605::SmR/SpR DNA cassette. Their sequences are provided in Table S2. C) UV-light image of the agarose gel showing that the Δslr0605::SmR/SpR mutant growing in presence, or absence (MM), of Sm and Sp has no WT chromosome. M = 1 Kb plus DNA Ladder (Thermo Scientific).
