# Supplementary material for: First Characterization of a Cyanobacterial Xi-Class Glutathione S-Transferase in Synechocystis PCC 6803
Source: Antioxidants (Basel). 2024 Dec 20;13(12):1577. doi: 10.3390/antiox13121577 (PMC11673678; doi:10.3390/antiox13121577)
Supplement: Supplementary file 1 [file antioxidants-13-01577-s001.zip › Fig S4.pptx]

## Slide 1
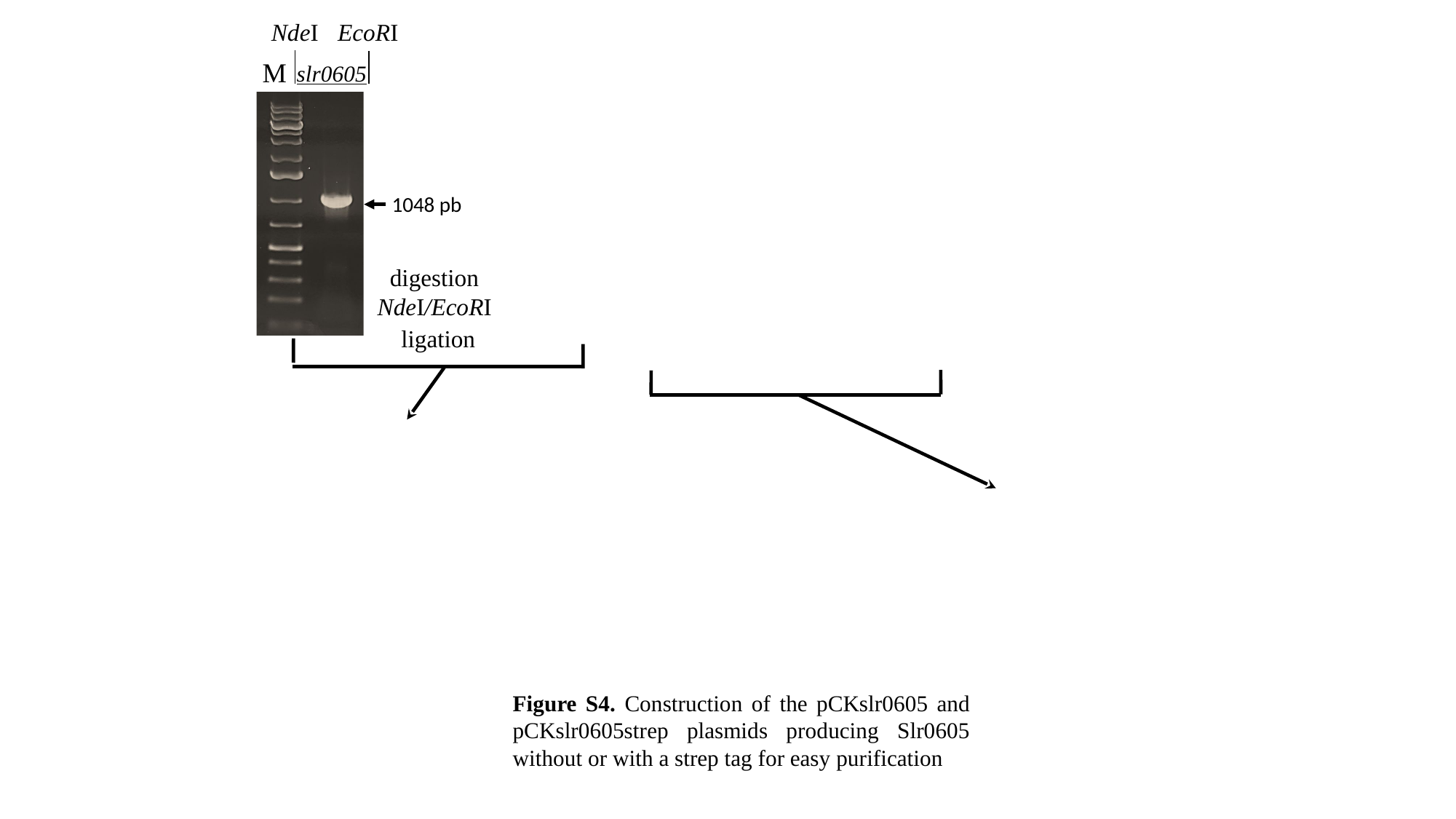

NdeI
EcoRI
M
slr0605
1048 pb
digestion NdeI/EcoRI
ligation
Figure S4. Construction of the pCKslr0605 and pCKslr0605strep plasmids producing Slr0605 without or with a strep tag for easy purification
