# Supplementary material for: First Characterization of a Cyanobacterial Xi-Class Glutathione S-Transferase in Synechocystis PCC 6803
Source: Antioxidants (Basel). 2024 Dec 20;13(12):1577. doi: 10.3390/antiox13121577 (PMC11673678; doi:10.3390/antiox13121577)
Supplement: Supplementary file 1 [file antioxidants-13-01577-s001.zip › Fig S5.pptx]

## Slide 1
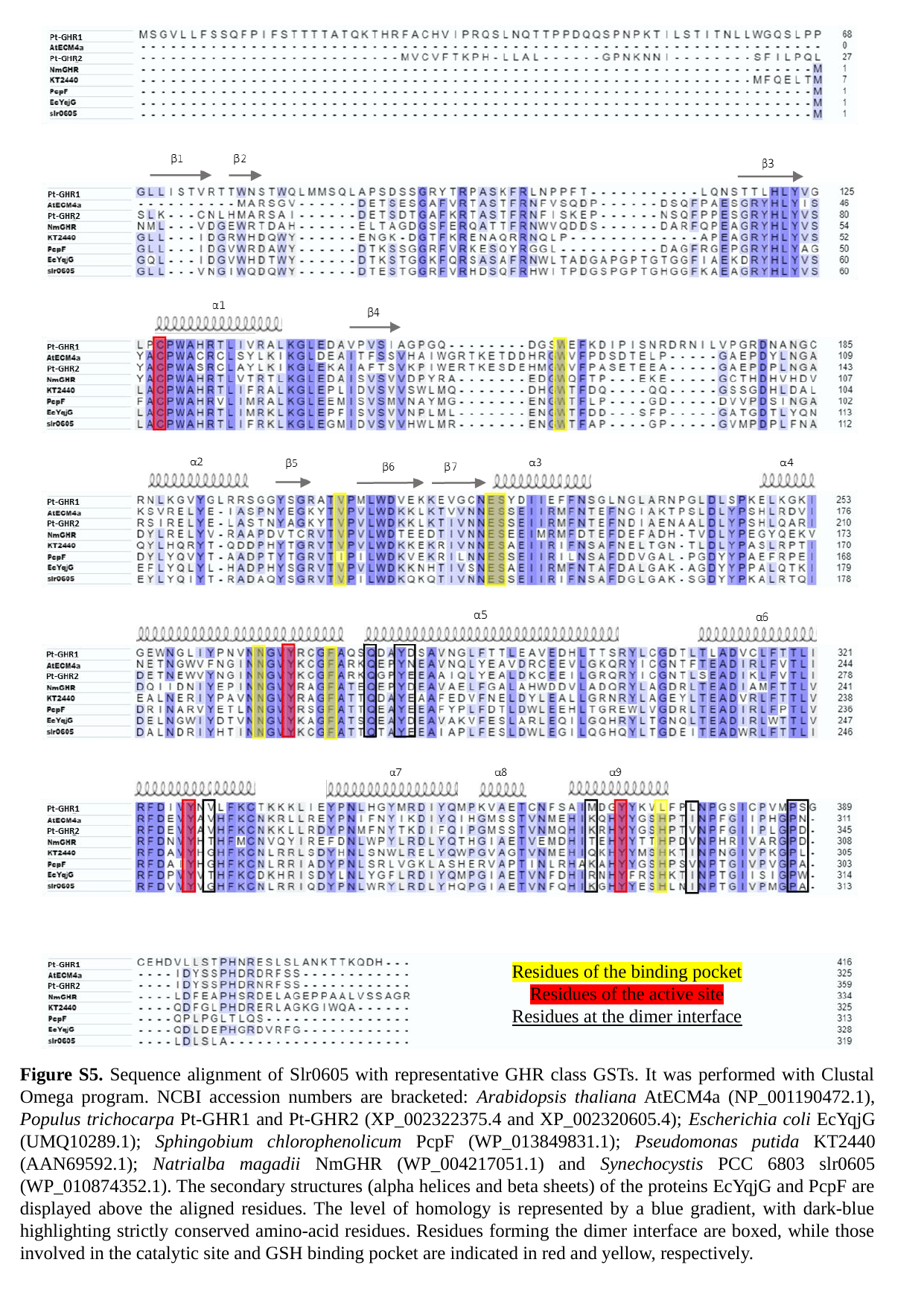

Residues of the binding pocket
Residues of the active site
Residues at the dimer interface
Figure S5. Sequence alignment of Slr0605 with representative GHR class GSTs. It was performed with Clustal Omega program. NCBI accession numbers are bracketed: Arabidopsis thaliana AtECM4a (NP_001190472.1), Populus trichocarpa Pt-GHR1 and Pt-GHR2 (XP_002322375.4 and XP_002320605.4); Escherichia coli EcYqjG (UMQ10289.1); Sphingobium chlorophenolicum PcpF (WP_013849831.1); Pseudomonas putida KT2440 (AAN69592.1); Natrialba magadii NmGHR (WP_004217051.1) and Synechocystis PCC 6803 slr0605 (WP_010874352.1). The secondary structures (alpha helices and beta sheets) of the proteins EcYqjG and PcpF are displayed above the aligned residues. The level of homology is represented by a blue gradient, with dark-blue highlighting strictly conserved amino-acid residues. Residues forming the dimer interface are boxed, while those involved in the catalytic site and GSH binding pocket are indicated in red and yellow, respectively.
