# Supplementary material for: First Characterization of a Cyanobacterial Xi-Class Glutathione S-Transferase in Synechocystis PCC 6803
Source: Antioxidants (Basel). 2024 Dec 20;13(12):1577. doi: 10.3390/antiox13121577 (PMC11673678; doi:10.3390/antiox13121577)
Supplement: Supplementary file 1 [file antioxidants-13-01577-s001.zip › Fig S6.pptx]

## Slide 1
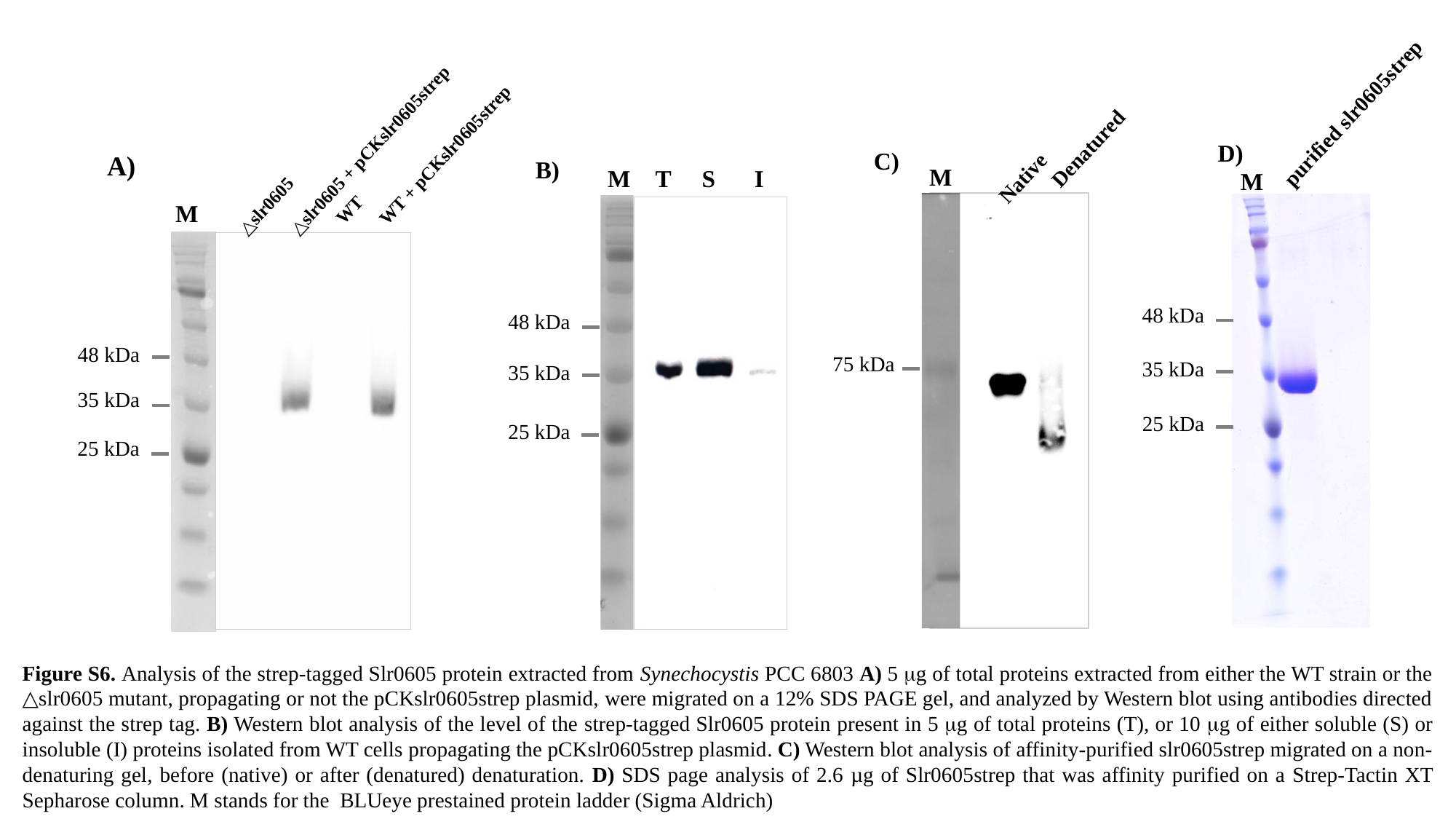

purified slr0605strep
Denatured
WT + pCKslr0605strep
△slr0605 + pCKslr0605strep
D)
C)
A)
B)
T
S
I
M
48 kDa
35 kDa
25 kDa
Native
M
M
WT
△slr0605
M
48 kDa
48 kDa
75 kDa
35 kDa
35 kDa
25 kDa
25 kDa
Figure S6. Analysis of the strep-tagged Slr0605 protein extracted from Synechocystis PCC 6803 A) 5 mg of total proteins extracted from either the WT strain or the △slr0605 mutant, propagating or not the pCKslr0605strep plasmid, were migrated on a 12% SDS PAGE gel, and analyzed by Western blot using antibodies directed against the strep tag. B) Western blot analysis of the level of the strep-tagged Slr0605 protein present in 5 mg of total proteins (T), or 10 mg of either soluble (S) or insoluble (I) proteins isolated from WT cells propagating the pCKslr0605strep plasmid. C) Western blot analysis of affinity-purified slr0605strep migrated on a non-denaturing gel, before (native) or after (denatured) denaturation. D) SDS page analysis of 2.6 µg of Slr0605strep that was affinity purified on a Strep-Tactin XT Sepharose column. M stands for the BLUeye prestained protein ladder (Sigma Aldrich)
