# Supplementary material for: First Characterization of a Cyanobacterial Xi-Class Glutathione S-Transferase in Synechocystis PCC 6803
Source: Antioxidants (Basel). 2024 Dec 20;13(12):1577. doi: 10.3390/antiox13121577 (PMC11673678; doi:10.3390/antiox13121577)
Supplement: Supplementary file 1 [file antioxidants-13-01577-s001.zip › Tables S1 and S2 .pdf]

Table S1. List of plasmids and strains used in this study

Strains/Plasmids	Relevant features	Reference
Escherichia coli CM404	*E. coli* strain containing the self-transmissible plasmid pRK2013 for conjugative transfer of RSF1010-derived plasmids in cyanobacteria	[34]
Escherichia coli TOP10	*E. coli* strain for cloning and conjugation	Invitrogen
Synechocystis sp. PCC6803	Best-studied unicellular cyanobacterium	Pasteur institute (Paris, France)
pUC4K	Vector used for PCR amplification of the Km^R cassette with no transcriptional terminator (TT)	Pharmacia
pGEM-T	Cloning vector	Promega
pΔslr0605::Sm^R/Sp^R	pGEMT harboring the Sm^R/Sp^R marker flanked by the up- and downstream regions of the *Synechocystis slr0605* gene. This plasmid was used to replace the *slr0605* coding sequence (CS) by the Sm^R/Sp^R marker.	This study
pΔsufR	pGEMT with a *SmaI* restriction site in between the up- and downstream regions of the *slr0088* (*sufR*) gene of *Synechocystis*	This study
pΔsufR::Km^R	pΔsufR harboring the Km^R marker in its *Sma*I site. This plasmid was used to replace the *Synechocystis sufR* CS by the Km^R marker.	This study
pTwist Amp medium copy + slr0605strep	pTwist harboring *slr0605* fused to the 8 aa Strep-Tag II CS	This study
pCK	RSF1010-derived plasmid vector (Km^R, Cm^R) harboring the strong pR promoter for constitutive gene expression in *E. coli* and cyanobacteria	This study
pCKslr0605	pCK derivative overexpressing *slr0605* in *Synechocystis*.	This study
pCKslr0605strep	pCK derivative overexpressing the *slr0605* CS fused to strep-tag sequence in *Synechocystis*.	This study

Abbreviation: TT; transcriptional terminator, ORF; Open reading frame, Δ; deletion.

[34] Mermet-Bouvier, P., and Chauvat, F. (1994) A conditional expression vector for the cyanobacteria *Synechocystis* sp. strains PCC6803 and PCC6714 or *Synechococcus* sp. strains PCC7942 and PCC6301. *Current Microbiology* **28**: 145–148.

Table S2 List of PCR primers used in this study

Name	**DNA Sequence (5' →3')**	**Use and the relevance features**
Deletion of the slr0605 gene		
pGEMt-R0605-FWa	tcccggccgccatggccgcgggattCCATGATTGTCCACCGGGATAAGGC	Forward and reverse primers for PCR amplification of the slr0605 upstream region & addition of sequences (lower cases) for Gibson-assembly cloning of the Sm^{R}/Sp^{R} deletion cassette of *slr0605* in the pGEMT plasmid
R0605-RVa	GTCCCTGCGCTGAAGATTGCC	
Sm/Sp-A0605-FW	ggcaatcttcagcgcagggacGCGCTCACGCAACTGGTCCA	Forward & reverse primers for PCR amplification of the Sm^{R}/Sp^{R} marker & addition of sequences (lower cases) for Gibson cloning with the upstream and downstream region of *slr0605* flanking a SmaI site
Sm/Sp-B0605-RV	CAGGAATTATTAGCCAAGctcccaatttgtgtagggct	
R0605-FWb	CTTGGCTAATAATTCCTGACGCTC	Forward and reverse primers for PCR amplification of the slr0605 downstream region & addition of sequences (lower cases) for Gibson cloning of the Sm^{R}/Sp^{R} deletion cassette of *slr0605* in pGEMT
pGEMt-R0605-RVb	caggcggccgcactagtgattCTGGGTTGCCTCCATCCACTCAATGTCAGCC	
Sm/Sp-FW	ATCTCGAACCGACGTTGCTG	Forward and reverse primers to verify chromosome segregation in the Δ*slr0605* mutant
Sm/Sp-RV	CCGACTACCTTGGTGATCTC	
SLR0605-FW	GGTGACAGTACCAATCCTTTGGGAC	
SLR605-RV	CGAATCAAGGTGGTAAATAAGCGCC	
Amplification and cloning of slr0605 CS in the pCK vector for strong constitutive expression of slr0605 or slr0605-strepII		
Amp_slr0605_For	CAGCGCAGGGACATTCATATGGGCTTACTCG	Forward & reverse primers for PCR amplification of the *slr0605* coding sequence (CS) to be cloned as a *Nde*1-*Eco*RI fragment in the pCK plasmid opened with the same enzymes
Amp_slr0605_Rev	TAGTATGAGCGTCAGGAATTCTTAGCCAAGGTGAGAT	
VerSeq_slr0605_For_1	CATCTTGTCTGCGACAGATT	Forward & reverse primers for PCR verification and DNA sequencing of the *slr0605* CS cloned in pCK
VerSeq_slr0605_Rev_1	ATGGTCTGCTTTTGTTTGTC	
VerSeq_slr0605_For_2	GCAGATGCCCAATATAGTG	
VerSeq_slr0605_Rev_2	ATGAAAACGTTTCAGTTTGC	
Seq_slr0605strep_For1	GTAAAACGACGGCCAGT	Forward and reverse primers for PCR verification and DNA sequencing of pCKslr0605 or pCKslr0605strep
Seq_slr0605strep_Rev1	CAGGAAACAGCTATGAC	
pFC1_Pra1_VBG	TCATAAATTGCTTTAAGGCG	

pFC1_sq_rv_Theo	CGTTTCAGTTTGCTCATGGA	Forward & reverse primers for verification of the *slr0605* CS cloned in between the *Nde*I-*Eco*RI sites of the pCK vector
Deletion of the sufRgene		
sufR-i-A1	TTTGTAGGGTTGGTTGACCA	Forward & reverse primers for PCR amplification of the *sufR* gene upstream region and addition of a *Sma*I restriction site (underlined)
sufR-i-R1 bis	CTTGAGTCCCGGGTTGTGGCCAGTAAACTGAAAGGACAACC	
sufRA2 bis	CCACAACCCGGGACTCAAGCCGAATCCTACCCCACCGT	Forward & reverse primers for PCR amplification of the *sufR* downstream region and addition of a *Sma*I site (underlined)
sufR-i-R2	TTGCACTCGTCTGGGTCGCC	
sufR-v-A1	CAGAAATGAGTCGCACCACG	Forward primer in the upstream region of *sufR* to verify the replacement of the *sufR* CS by the Km^R cassette in the Δ*sufR* mutant
KmR9	CGCGGCCTCGAGCAAGACGTTTCCCGTTGAATATGGCTC	Reverse primer in the Km^R marker to verify the presence of the Km^R cassette in the deletion plasmid and the Δ*sufR* mutant
KmA9	GCTCTCATCAACCGTGGCTCCCTCACTTTCTGG	Forward primer in the Km^R cassette to verify the replacement of the *sufR* CS by the Km^R marker in the Δ*sufR* chromosome
sufR-v-R1	CAGTTGTTTAAGCAGGAACC	Reverse primer in the *sufR* downstream region to verify the replacement of the *sufR* CS by the Km^R cassette in the Δ*sufR* mutant
sufR-A1	ACGTTACGGTGAATTTGCCC	Forward primer in *sufR* gene to verify the presence of WT chromosome copies
SufR-R1	TTCAGCCATGGCGATCGCCG	Reverse primer in the *sufR* gene to verify the presence of WT chromosome copies
